# Editorial: Actinobacteria: Recent Trends in Genomics, Omics Study and Discovery of Novel Natural Products

**DOI:** 10.3389/fmicb.2021.799737

**Published:** 2021-12-08

**Authors:** Ajit Kumar Passari, Sergio Sánchez

**Affiliations:** Departamento de Biología Molecular y Biotecnología, Instituto de Investigaciones Biomédicas, Universidad Nacional Autónoma de México, México, Mexico

**Keywords:** actinobacteria, genomics, natural bioactive compounds, transcriptomics, metabolomics

Bacteria, particularly actinobacteria, and fungi, among the microorganisms that generate the constituents above, produce a varied array of small bioactive molecules with significant potential for use in medicine (O‘Brien and Wright, 2011). Antibiotics, pigments, growth hormones, anticancer drugs, and other microbial secondary metabolites are not essential for microbe growth and development, but they have shown considerable promise for human and animal health (Sánchez and Demain, 2014). These secondary metabolites are formed mainly by the activation of cryptic gene clusters that are inactive under certain conditions; consequently, increasing the expression of these clusters could assist in exploiting microorganism's chemical variability (Pettit, 2011; Guzmán-Trampe et al., 2017; Xu et al., 2019).

Actinobacteria are Gram-positive microbes with rod-shaped or filamentous morphology. They inhabit diverse environments and have a high G + C content in their DNA. Actinobacteria have had a significant influence on human health and well-being (Demain and Sanchez, 2009). Actinobacteria have been obtained from various ecosystems like saline soil, freshwater sediments, sponges, animal and human guts, medicinal plants, deep forests, hot springs, etc. Due to their various ecological functions have multiple applications in agriculture, biomedical, industrial, and pharmaceutical, apart from antibiotic production (Demain et al., 2019). The application of omics tools (genomics, proteomics, transcriptomics, metabolomics, and NGS studies) in actinobacteria has impacted novel bioactive compounds detection and production (Orsi et al., 2016; Singh et al.). Still, the physiological functions of actinobacteria and their ecological interactions await further investigation. The publication of the complete genome sequence of the actinobacteria model *Streptomyces coelicolor* A3 (2) stimulated the development of computational resources. These include bioinformatics tools like “Antibiotics and Secondary Metabolites Analysis SHell (antiSMASH)” and the “Prediction Informatics for Secondary Metabolomes (PRISM)” (Martínez-Klimova et al., 2016). With these instruments, many researchers have discovered the presence of “cryptic” or silent biosynthetic gene clusters (Takagi and Shin-ya, 2011; Harrison and Studholme, 2014). Therefore, many researchers have used the CRISPR/Cas9 system as a tool to elicit the production of unknown secondary metabolites by (a) either knock-in genes to activate silent biosynthetic gene clusters or (b) deleting repressor genes (Jia et al., 2017; Zhang et al., 2017).

Under the title “Actinobacteria: Recent Trends in Genomics, Omics Study and Discovery of Novel Natural Products,” a total of six articles were published, covering a variety of topics orbiting around actinobacteria. The special issue includes three reviews and three research articles on actinobacteria dealing with: antibiotic production, ecological modifications, transcriptomic profiling, gene modulation by post-translational modifications, and the discovery of novel secondary metabolites with their bioactive properties. Thus, a survey by Droste et al. emphasized that transcriptome and proteome data are valuable for improving the annotation of the strain *Streptomyces lividans* TK24 genome. Strain TK24 could be used to examine secondary metabolite gene clusters analysis. Martin et al. came out with the role of post-translational modifications in bacterial metabolism modulation, especially on their effects on secondary metabolite biosynthesis. Kim et al. have focused on enhanced ohmyungsamycin A (OMS) production through adenylation (A2) domain engineered *Streptomyces* strains under the optimized culture conditions. Ohmyungsamycin A showed significant activity against *Mycobacterium tuberculosis* and human cancer cells. Hesketh et al. pointed out that results of chemotranscriptomic profiling defined the specific signatures of the glycopeptide antibiotics dalbavancin, vancomycin, and chlorobiphenyl-vancomycin tested at the bacterium's transcriptional response level. Singh et al. described that actinobacteria are well-known for producing diverse secondary metabolites and supported the genomic approach developments as a gateway for examining and manipulating novel antibiotic gene clusters. Kalam et al. described the influence of the phylum acidobacteria in vital ecological processes. They proposed exploring these bacteria's genetic attributes of understand better the functions and ecological significance in the soil-plant environment.

We are enchanted to present this research topic in Frontiers in Microbiology. We hope this special issue will be exciting and beneficial to the journal's readers and spread the information of actinobacteria importance in diverse areas. The knowledge accessible above is promising but still limited. Finally, we acknowledge all contributors, totaling 40 authors, for the relevant scientific information within the research articles and reviews compiled in this issue. We are pretty sure that the information covered and presented will be fascinating and convenient for the readers and can be the idea for the investigation on “Actinobacteria: Genomic approach for the production of Natural Products.”

## Author Contributions

AP and SS organized this topic and wrote the editorial article for publication. All authors contributed to the article and approved the submitted version.

## Conflict of Interest

The authors declare that the research was conducted in the absence of any commercial or financial relationships that could be construed as a potential conflict of interest.

## Publisher's Note

All claims expressed in this article are solely those of the authors and do not necessarily represent those of their affiliated organizations, or those of the publisher, the editors and the reviewers. Any product that may be evaluated in this article, or claim that may be made by its manufacturer, is not guaranteed or endorsed by the publisher.
